# Agenesis of the Left Upper Lobe of the Lung in a Young Adult Male: A Case Report

**DOI:** 10.7759/cureus.83412

**Published:** 2025-05-03

**Authors:** Vijay T Salve, Jay Manchanda, Osheen Singhal, Anushri Kaul Gupta, Subhash Chand Choudhary

**Affiliations:** 1 Respiratory Medicine, ESIC Medical College and Hospital, Alwar, IND; 2 Cardiology, ESIC Medical College and Hospital, Alwar, IND; 3 Radiodiagnosis, ESIC Medical College and Hospital, Alwar, IND

**Keywords:** agenesis, congenital anomaly, ct pulmonary angiogram, left upper lobe of lung, lung development

## Abstract

Left upper lobe agenesis is an exceedingly rare condition, and the patients usually remain asymptomatic in their childhood. We report a case of a 19-year-old male who presented with nonspecific chest pain and shortness of breath after running. On investigation, we found that he had haziness on the left upper and mid zones and blunting of the left costophrenic angle on chest X-ray posteroanterior (PA) view. The patient had no history of pleuro-pulmonary tuberculosis or any thoracic surgery. Further workup with CT thorax and CT pulmonary angiogram (CTPA) revealed a complete absence of the left upper lobe with herniation of the right lung to the left hemithorax and absence of the left superior lobar pulmonary artery. He was managed conservatively without any complications.

## Introduction

Left upper lobe agenesis is a rare congenital anomaly characterized by the complete absence of the upper lobe and its associated structures. The prevalence of this condition is extremely low, estimated to be as low as 0.0034% (a record from Louisville Hospital, from 1948 to 1967, showed congenital maldevelopment of lungs in four cases among 114,569 admissions) [1]. Due to its typically asymptomatic nature, diagnosis is often delayed and only occurs incidentally during imaging studies performed during diagnostic workup. Clinical presentations vary, with some patients remaining asymptomatic throughout their lives, while others may experience exertional breathlessness, recurrent respiratory infections, or chronic cough. Previous reports have identified cases where agenesis was detected during evaluation of pulmonary tuberculosis [2], mucoepidermoid carcinoma [3], and abnormal cardiac auscultatory findings [4].

Left upper lobe agenesis has been found in association with cardiovascular (e.g., patent ductus arteriosus and patent foramen ovale), genitourinary, musculoskeletal, and gastrointestinal anomalies [5,6]. The first reported case of left lung agenesis was documented by De Pozze in 1673, during the autopsy of a young female [7]. Muhamed (1923) identified a similar case during a medicolegal autopsy in India [8]. More recently, a case of agenesis of the left upper lobe associated with patent foramen ovale with patent ductus arteriosus was reported by Gowrinath et al. [9]. Another case of left upper lobar agenesis was reported by Gupta et al. in 2017 [10]. Given the rarity of the condition, we believe a case report like the present one plays a crucial role in increasing awareness among clinicians and improving diagnostic accuracy.

## Case presentation

A 19-year-old male student with no history of substance use presented to the pulmonology outpatient department with complaints of generalized dull aching chest pain, which was non-radiating with no aggravating or relieving factor for 15 days. He also reported shortness of breath after running since childhood, which had not progressed over the years. However, the patient did not experience shortness of breath while hurrying on level ground. There was no history of pulmonary tuberculosis or lung surgery in the past. He had been hospitalized for “pneumonia” in childhood. There was no other remarkable history. His vital parameters were within normal limits (heart rate: 64 bpm, blood pressure: 124/64 mmHg, SpO_2_: 99 on room air).

**Figure 1:**
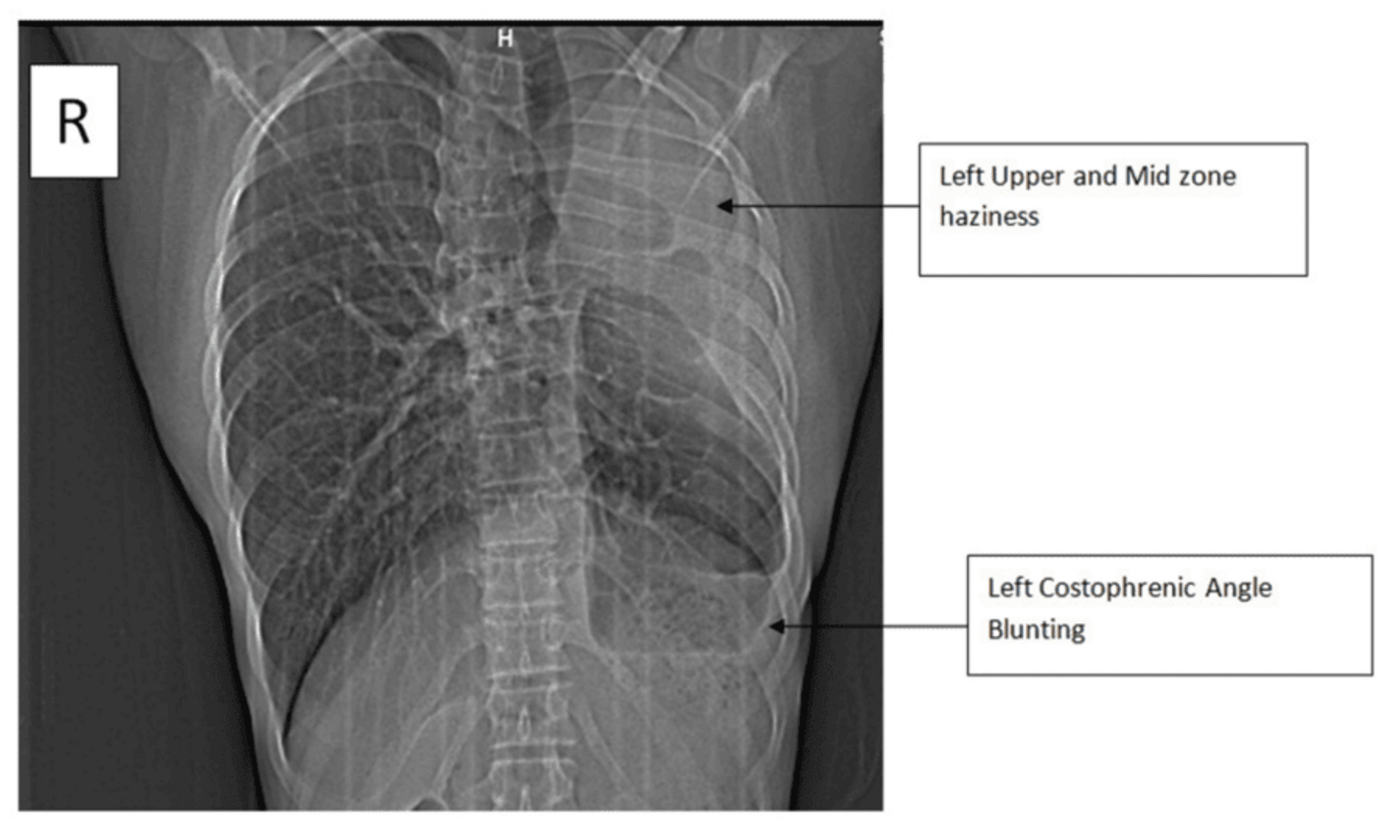
Chest X-ray posteroanterior view The image shows left upper and mid zone haziness and left costophrenic angle blunting (black arrows)

**Figure 2:**
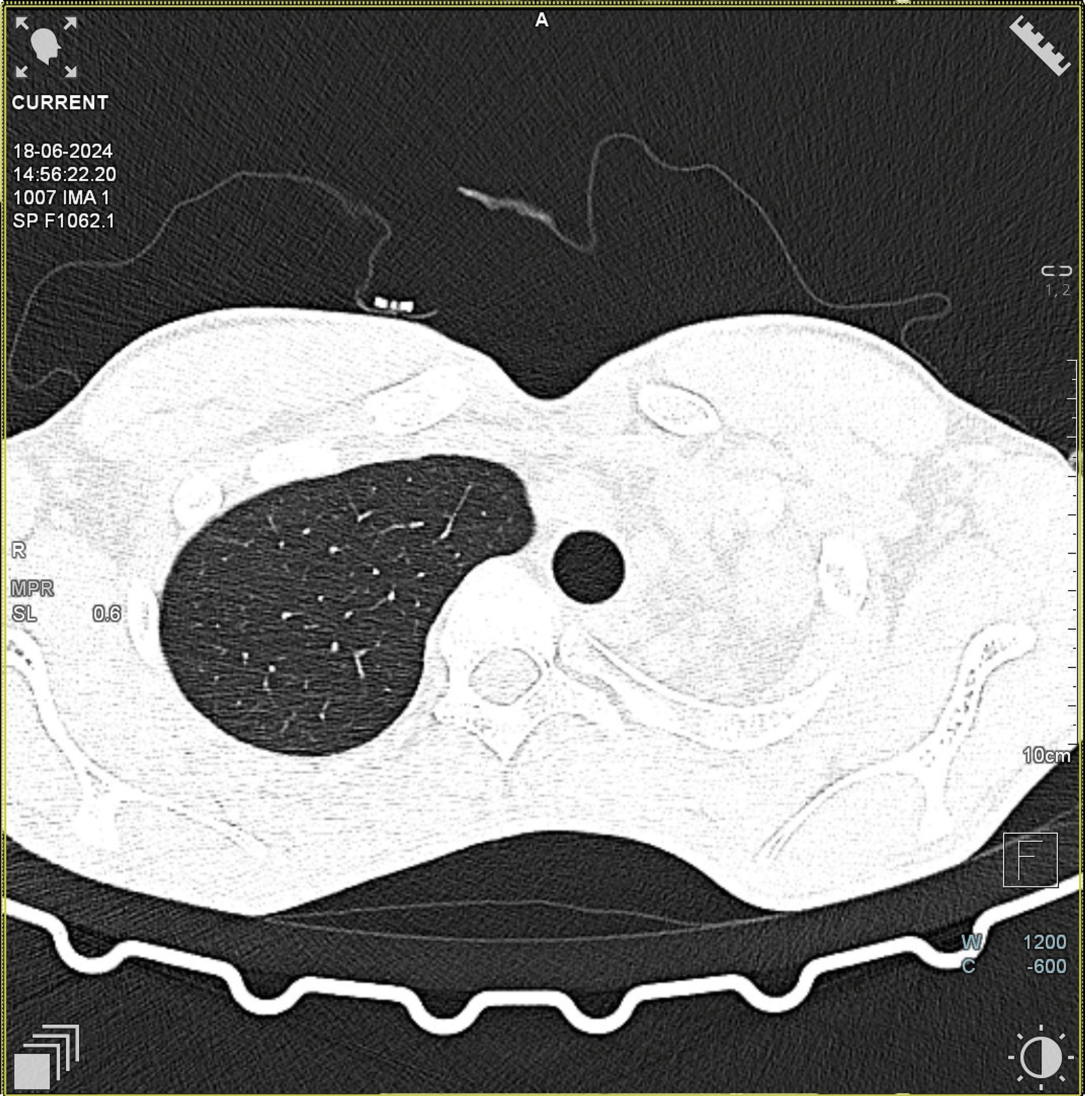
CT chest-axial section at T3 level The image shows the absence of the left upper lobe CT: computed tomography

Respiratory system examination revealed that the patient had pectus excavatum. Breath sounds in the left mammary area, the left axillary area, and the interscapular area on the left side were diminished. Adventitious sounds were not heard. Cardiovascular system examination showed that the apex impulse was shifted to the anterior axillary line at the fourth intercostal space. S1S2 were heard normally. Chest X-ray showed homogeneous haziness in the left upper and mid zones with blunting of the left costophrenic angle, suggestive of pleural thickening (Figure 1). Subsequently, the patient was further investigated. CT thorax was ordered, confirming the absence of the left upper lobe (Figure 2).

**Figure 3:**
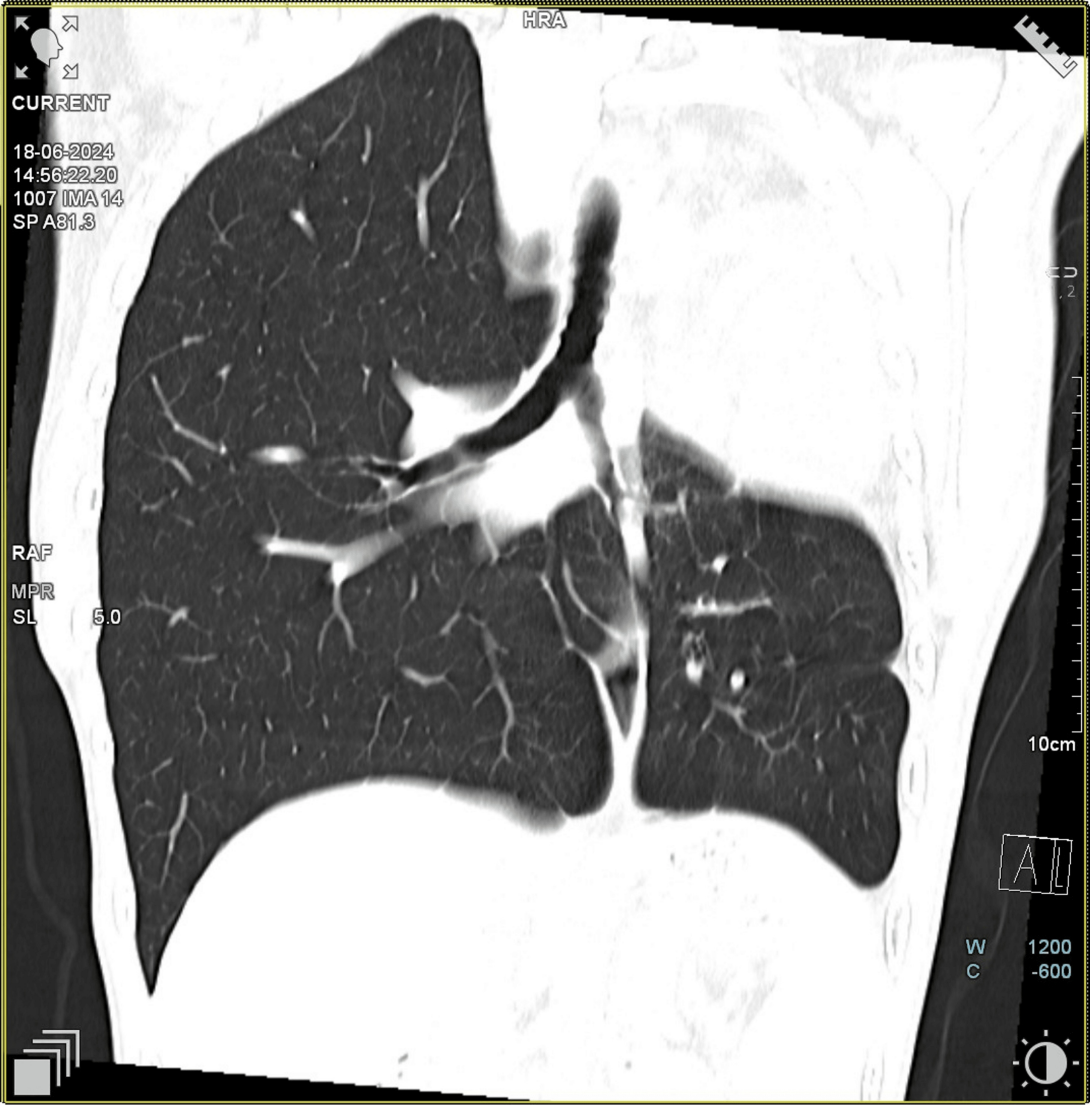
CT thorax coronal section The image shows the absence of the left upper lobe bronchus and the left upper lobe. The left main bronchus continues as the left lower lobe bronchus CT: computed tomography

**Figure 4:**
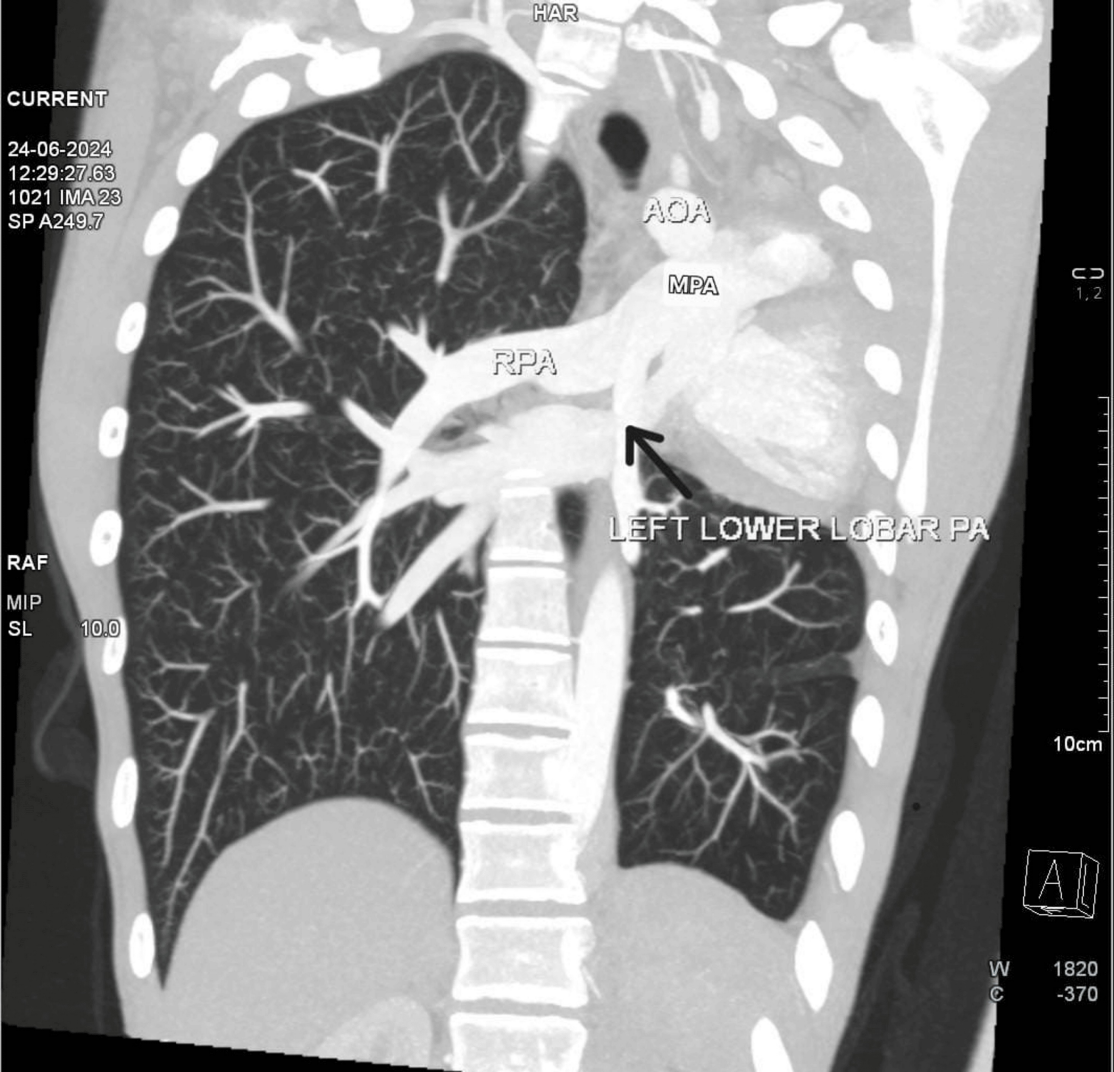
CT pulmonary angiogram CT pulmonary angiogram is suggestive of displaced heart and great vessels in empty space due to the absent left upper lobe. The image shows the left inferior lobar pulmonary artery branching from the main pulmonary artery (black arrow) CT: computed tomography

CT chest coronal section was suggestive of left main bronchus continuing as left lower lobe bronchus with herniation of the right lung to the contralateral side (Figure 3). The patient's 2D echo was suggestive of normal cardiac chamber dimensions and relations, absent left upper lobar branch of the pulmonary artery, and trivial MR. A CT pulmonary angiogram (CTPA) (Figure 4) was performed as part of further investigations, which revealed the absence of the left upper lobe and shifting of the heart and major vessels to the empty space due to the absence of the left upper lobe. The inferior lobar pulmonary artery was visualized in CTPA.

The patient's spirometry was suggestive of a restrictive ventilatory defect. Forced vital capacity (FVC) was 70.4% of predicted, and forced expiratory volume in the first second (FEV1)/FVC ratio was 80.3% of predicted. ECG was suggestive of incomplete right bundle branch block (RBBB).

## Discussion

Agenesis of the left upper lobe of the lung is an extremely rare condition, and patients are usually asymptomatic in childhood. They may present with exertional dyspnea, as in our case. This delayed presentation leads to delayed diagnosis, as symptoms are nonspecific and might be mistakenly attributed to other respiratory conditions. Our patient had a history of exertional dyspnea, occasional cough with scanty mucoid expectoration, and, upon further examination, a chest wall deformity, pectus excavatum, was noted. Chest X-ray showed opacity in the left upper and mid zones with left CP angle blunting. Pulmonary function testing revealed restrictive ventilatory defects, with the FVC at 70% of the predicted value. Such findings support the diagnosis of a restrictive lung disease. CTPA is crucial for confirming the diagnosis and excluding other conditions such as cardiac anomalies. [11]. The CTPA showed shifting of the heart and great vessels into the space created by the absence of the left upper lobe, with an absent upper lobar pulmonary artery. The inferior lobar pulmonary artery originated from the main pulmonary artery.

Lung development begins in the fourth week of gestation from the ventral wall of the foregut. A laryngotracheal groove develops from the ventral wall of the pharynx and divides into right and left lung buds on the 28th day of gestation. Lung development is divided into five phases: embryonic, pseudo-glandular, canalicular, saccular, and alveolar [10]. Any factor affecting the development of the lung bud can result in malformation, like agenesis and hypoplasia of the left upper lobe, any other lobe, or the entire lung. Genetic factors, viruses, vitamin A deficiency during pregnancy, and intake of drugs like allopurinol (teratogenic effect) have been implicated as etiological factors in the maldevelopment of the lungs [12,13].

Spencer and Snider classified the malformation of the lung into three groups. It was further modified by Boyden [14], as follows: type 1 (agenesis) - the complete absence of lung and bronchus and no vascular supply to the affected side; type 2 (aplasia) - rudimentary bronchus with complete absence of pulmonary parenchyma; and type 3 (hypoplasia) - the presence of variable amounts of bronchial tree, pulmonary parenchyma, and supporting vasculature.

Our patient had a complete absence of lung parenchyma and bronchus, and the superior branch of the left pulmonary artery was absent. History of lobectomy or pneumonectomy, fibrothorax, and segmental or lobar collapse may be considered as differential diagnoses in this condition. Most patients with partial lung agenesis require conservative management focusing on addressing respiratory symptoms. Our patient was managed conservatively, as he did not have an infection or hypoxia. Regular follow-up and lung function monitoring are required in such patients. Pulmonary rehabilitation and vaccination against common viral and bacterial respiratory infections should be considered in such cases as part of long-term management.

## Conclusions

Left upper lobe agenesis should be considered as one of the differential diagnoses in cases of left upper zone persistent opacity and mediastinal shift on chest X-ray, as it remains asymptomatic or may manifest some respiratory symptoms. A thorough history, CT thorax, and CTPA are crucial for diagnosis and to differentiate it from other conditions such as fibrothorax and post-thoracic surgical changes. As a part of long-term management in such cases, it is advisable to monitor lung function. Pulmonary rehabilitation and vaccination against common viral and bacterial infections should be considered as well.
